# The Potential use of a Curcumin-Piperine Combination as an Antimalarial Agent: A Systematic Review

**DOI:** 10.1155/2021/9135617

**Published:** 2021-10-11

**Authors:** Shafia Khairani, Nisa Fauziah, Hesti Lina Wiraswati, Ramdan Panigoro, Endang Yuni Setyowati, Afiat Berbudi

**Affiliations:** ^1^Doctoral Program in Medical Science, Faculty of Medicine, Padjadjaran University, Bandung, Indonesia; ^2^Veterinary Medicine Program, Faculty of Medicine, Padjadjaran University, Bandung, Indonesia; ^3^Department of Biomedical Sciences, Parasitology Division, Faculty of Medicine Universitas Padjadjaran, Bandung, Indonesia; ^4^Department of Biomedical Sciences, Biochemistry and Molecular Biology Division, Faculty of Medicine Universitas Padjadjaran, Bandung, Indonesia

## Abstract

Malaria remains a significant global health problem, but the development of effective antimalarial drugs is challenging due to the parasite's complex life cycle and lack of knowledge about the critical specific stages. Medicinal plants have been investigated as adjuvant therapy for malaria, so this systematic review summarizes 46 primary articles published until December 2020 that discuss curcumin and piperine as antimalarial agents. The selected articles discussed their antioxidant, anti-inflammatory, and antiapoptosis properties, as well as their mechanism of action against *Plasmodium* species. Curcumin is a potent antioxidant, damages parasite DNA, and may promote an immune response against *Plasmodium* by increasing reactive oxygen species (ROS), while piperine is also a potent antioxidant that potentiates the effects of curcumin. Hence, combining these compounds is likely to have the same effect as chloroquine, that is, attenuate and restrict parasite development, thereby reducing parasitemia and increasing host survival. This systematic review presents new information regarding the development of a curcumin-piperine combination for future malaria therapy.

## 1. Introduction

Malaria is still a significant health problem, with more than 220 million people affected and millions of deaths annually worldwide, particularly children and pregnant women [1]. The current availability of antimalarial drugs in reducing malaria morbidity and mortality in endemic areas does not positively impact. Still, it creates new problems, such as the emergence of drug-resistant parasites [2, 3]. This is a significant challenge to human health; consequently, new antimalarial drugs or treatment strategies are urgently needed. However, developing an effective antimalarial drug is challenging due to the complex life cycle of parasite. Plasmodia infection begins with an asymptomatic liver-stage, followed by symptomatic blood-stage infection [2, 4]. Most studies have shown that protective immunity will automatically develop against blood-stage infections, after repeated exposure to the parasites, but it remains unclear at the liver stage [2, 5, 6]. Furthermore, it becomes more challenging to fortify the host when the parasites enter the blood-stage without being interfered at the liver-stage; consequently, the load of the parasite in the blood could be unruly high [2].

The use of medicinal plants can modulate the immune response, which significantly impacts health [2–7]. For example, in India, mostly Indians consume foods containing spices/herbs, such as garlic, ginger, turmeric, and black pepper, which are known to have antimalarial activity [8–11]. Turmeric (*Curcuma longa*) is an ancient spice from Southeast Asia, used as a dye and a condiment [12]. It is one of the cheapest spices globally and has been used for 4,000 years to treat various ailments [12, 13]. It contains an active substance, curcumin *(bis-α, β-unsaturated β-diketone)*, commonly known as *diferuloylmethane*, which has a broad spectrum of biological and pharmacological activities, including antioxidant [14], anti-inflammatory [15], antimicrobial, and anticarcinogenic [14] properties. Additionally, the hepato- and nephroprotective [16, 17], thrombosis suppression [18], myocardial infarction protective [19], hypoglycemic [20], and antirheumatic [21] effects of curcumin are also well established. Curcumin exhibits potent activity against other parasites including *Leishmania* [22], *Cryptosporidium parvum* [23], *Schistosoma mansoni* [24], *Giardia lamblia* [25], and *Trypanosoma cruzi* [26]. Moreover, it has been shown to possess antimalarial activity against various *Plasmodium* species *in vitro* [27–31]. Similar to turmeric, black pepper (*Piper nigrum*) is also used as a traditional antimalarial medicine in Calabria (South Italy) and India, especially for treating malaria with symptoms of periodic fever and hepatomegaly [32, 33]. It is also an ancient spice from the coast of Malabar in India, which contains an active substance called piperine (chemically, *piperoylpiperidine*), which has been used to treat cholera, flatulence, arthritis, digestive disorders, asthma, and cancer [34–37].

Many studies (*in vitro, in vivo*, as well as clinical trials) have described the use of curcumin and piperine as antimalarial drugs, either alone or combined with current antimalarial drugs [10, 28, 30, 38–40]. However, no studies have discussed the potential use of curcumin-piperine combinations and their possible mechanisms of action. This systematic review summarizes the use of curcumin and piperine, identifies their possible antimalarial mechanisms, and determines the role of curcumin-piperine in the management of malaria. For the future, this study can be used as a reference to produce a potential antimalarial agent.

## 2. Materials and Methods

### 2.1. Literature Search Strategy

Two electronic databases, i.e., Google Scholar and PubMed, were searched for relevant studies published between 1995 and December 2020. The search terms used for this systematic review included “curcumin, curcuma, malaria” or “piperine, piper nigrum, malaria.” The language was restricted to English.

### 2.2. Eligibility Criteria

#### 2.2.1. Inclusion Criteria

All articles published in English language between 1995 and December 2020 in any setting with an aim of finding the potential use of curcumin or piperine for malaria regardless of the *Plasmodium* species whether *P. falciparum, P. vivax, P. berghei, P. chabaudi*, or *P. yoelii.*

#### 2.2.2. Exclusion Criteria

**Figure 1:**
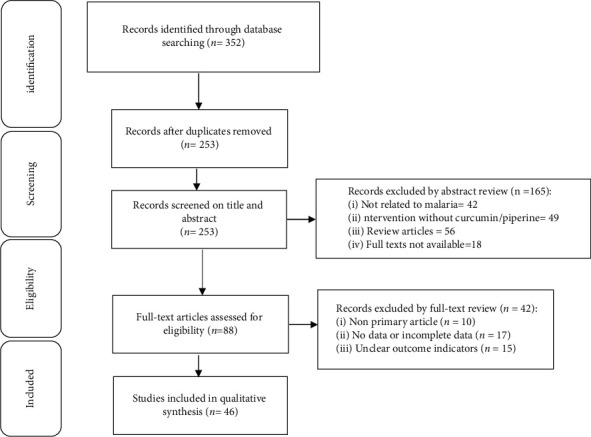
Flow chart of literature selection.

Studies of curcumin or piperine in malaria do not provide complete data or unclear outcome indicator. Review articles, case reports, letter to the editor, conference papers, and articles published in languages other than in English. Full texts are not accessible/irretrievable. The systematic review was guided by the PRISMA guidelines. The PRISMA diagram detailing the selection process is shown in Figure 1.

### 2.3. Study Selection and Data Extraction

For this systematic review, two researchers independently read the title and abstract for screening, continued by reading the full text of the research study and performing literature screening and data extraction according to inclusion and exclusion criteria. Disagreements of two researchers will be resolved by involving the third researcher to make final decision. The following data were extracted: year of publication, first author, type of study, subject, intervention characteristics (i.e., dosage and compound`s activities), and outcome measures.

### 2.4. Data Analysis

Due to the heterogeneity of the included studies, a meta-analysis was not conducted. Data analysis was performed descriptively using Microsoft Excel 2019. Data analysis was presented in a narrative form.

## 3. Results and Discussion

### 3.1. Selection Studies

A total of 352 articles were obtained according to the search strategy. We acquired the remaining 253 articles after removing duplicates and were subsequently filtered by title and abstract so that 165 studies were excluded. The remaining 88 articles were further screened by reading the full-text articles, and 42 articles were excluded. Finally, this review includes 46 qualitative studies.

### 3.2. Curcumin as an Antiplasmodium

In total, 46 primary articles were identified and 41 articles discussed curcumin (Table 1), reporting that curcumin exerts antiplasmodium effects through various activities/mechanisms. The pathogenesis of malaria is multifactorial involving the complex life cycle of the parasites. During a blood meal, a malaria-infected mosquito inoculates sporozoites (SPZ) into the human skin, enter the liver via bloodstream, and infect hepatocytes. At the liver-stage (exoerythrocytic), SPZ produce thousands of infective merozoites, enter the bloodstream, and infect the red blood cells (RBCs) to recruit the erythrocytic cycle that is responsible for clinical sign of the disease [75]. The infection level correlates with the parasite burden that elicits a defense mechanism to prevent the parasite from multiplying [30]. Curcumin (turmeric) acts as a prooxidant and antioxidant to modulate the innate immune response through the production of intracellular reactive oxygen species (ROS) for the clearance of parasites. ROS enhances the scavenger expression of the CD36 receptor on monocytes or macrophages, which mediates phagocytosis of the nonopsonization parasite-infected erythrocyte by macrophages [30, 42]. Also, curcumin promotes the immune response through induction of ROS production, resulting in the activation of PPARɣ/Nrf2 and upregulation of CD36 expression in monocytes/macrophages that recital the parasiticidal activity on the blood-stage parasite when administered in mice [30, 72]. ROS production can also be caused by the accumulation of large amounts of free heme, known as *ferriprotoporphyirin* [13], inducing oxidative stress which leads to parasite death. In this case, the parasite requires a free heme detoxification process by changing it to a nontoxic, inert, insoluble, crystal pigment, and blackish-brown form called hemozoin or *β*-hematin [76]. The formation of *β*-hematin is considered a key mechanism for heme detoxification in *Plasmodium* [76, 77]. The study conducted by Padmanaban et al. demonstrated that the curcumin-artemisinin combination inhibited hemozoin formation and increased ROS production in mice infected with *P. berghei*. The result suggests that the combination of these compounds is synergistic and results in optimal efficacy. Furthermore, *in vitro*, curcumin 0.4 mM exhibits an inhibitory effect on the formation of *β*-hematin, with an efficiency of 78.8% compared to amodiaquine (91.8%) and DMSO (10.7%) [13]. Similar findings were also obtained by Akhtar et al. [29], who reported that curcumin bound to chitosan nanoparticles cured rats of *P. yoelii* infection and inhibited the synthesis of *β*-hematin *in vitro* at IC50 (122 *μ*M ± 2.7). Curcumin bound to chitosan nanoparticles could increase bioavailability and metabolic stability. Some antimalarial drugs, i.e., chloroquine and amodiaquine, inhibit hemozoin formation in food vacuoles, preventing the detoxification of the released heme, thereby killing the parasites. Curcuminoid isolate has a similar role to chloroquine, so the interaction between ferriheme and curcumin is likely to allow the interaction of the Fe^3+^ metal center with one of the carbonyl groups on curcumin. Furthermore, the side-chain carboxyl group of heme will interact with one of the hydroxyl groups of curcumin [3]. The capability of curcumin as a prooxidant is also known to trigger the production of ROS, resulting in mitochondrial and core DNA damage and triggering pH changes in organelles that cause parasite death [42]. Furthermore, curcumin-induced hypoacetylation occurs on H3 in K9 and K14; nevertheless, not on H4 in K5, K8, K12, and K16. The result prompts us to think that curcumin can cause inhibition of the HAT PfGCN5 involved in parasite chromatin modifications [42]. Chromatin is a pivotal component of various cellular processes such as DNA transcription, replication, and repair [78]. It is composed of a nucleosome containing two copies of histones H2A, H2B, H3, and H4 which play a role in the epigenetic regulation of gene expression. Histone lysine acetylation is catalyzed by histone acetyltransferases (HATs), and it is eliminated by histone deacetylases (HDACs). Previous studies revealed that histone acetylation has great potential as a new therapeutic target. To date, several HDAC inhibitors have also been clinically tested for anticancer therapy [79]. *P. falciparum general control nondepressed 5* (PfGCN5) is a HAT that acetylates K9 and K14 from H3 histone. Drugs that impact on HDACs and impede histone acetylation in parasites have powerful antiparasitic actions. Curcumin serves as a HAT p300/CREB-binding protein (GST) inhibitor, but its inhibitory effect is selective because curcumin does not suppress the P300-associated factor of GNAT (*GEN5-related acetyltransferase*), a member of the HAT superfamily. Cui et al. [78] revealed that curcumin specifically inhibits PfGCN5 *in vitro* and has a cytotoxic effect against the parasite. Curcumin (5 *μ*M) is also reported to disrupt cellular microtubules of *Plasmodium* through depolarization of the microtubules during mitosis due to elevated ROS and is more prominent in the second cycle [31], similar to the effect of the microtubule-destabilizing drug vinblastine on *P. falciparum*. Molecular docking predicts that curcumin might bind to the alpha-beta tubulin heterodimer interface leading to altered microtubule morphology. This is supported by drug combination trials with antagonistic interactions between curcumin and colchicine which show competition for the same binding site. Alternatively, it is possible that curcumin does not bind directly to tubulin but is involved in global cell damage or due to the targeted effect of curcumin. Impaired microtubules inhibit cellular functionality, including apicoplast morphology [31, 80]. Previous studies regarding the effect of curcumin on *Plasmodium* viability have also been reported. Reddy et al. [27] revealed that curcumin (IC_50_ of 5 mM) inhibits the development of *P. falciparum* via PfATP6, the orthologue parasite on the SERCA (*sarcoplasmic-endoplasmic reticulum Ca*^*2+*^*- ATPase*) mammalian cells. Curcumin inhibits Ca^2+^-ATPase by stimulating a conformational change, which impedes the ATP from attachment. In this case, curcumin has the same activity as artemisinin [27]; thus, it is hypothesized that curcumin can decrease *Plasmodium* viability and reduce blood parasitemia, significantly increasing the survival rate.

Malaria is a highly inflammatory disease that requires drugs that can suppress the inflammatory response. Curcumin (therapeutic and prophylactic) can reduce TNF-*α* and IFN-*γ* (proinflammatory cytokines), increase IL-10 and IL-4 (anti-inflammatory cytokines), as well as modulate inflammatory cytokines mediated by inhibition of GSK3*β* (serine/threonine kinase which functions in glycogen metabolism and is the target of malaria therapy) [73]. Furthermore, sequestration is a pathological hallmark of *P. falciparum* infection, where erythrocytes can attach to the endothelium of vital organs in an attempt by the malaria parasite to evade the immune system [81]. The sequestration process can occur in both infected and uninfected erythrocytes due to lack of microvascular flow, causing damage to the blood-brain barrier, cerebral edema, and tissue hypoxia [30]. Sequestration is also recognized as a consequence of the expression of adhesion molecules (mostly ICAM1, VCAM1, and E-selectin) in brain endothelial cells induced by excessive production of inflammatory cytokines or by direct attachment of *P. falciparum* [82]. *In vitro* studies show that inflammation through the expression of ICAM1 results from *P. falciparum* adhesion to brain endothelial cells [30]. Curcumin can effectively control the inflammatory cascade due to the host immune response in cerebral malaria via the modulation of NF-*κ*B (*nuclear factor κ beta*), which plays an essential role in malaria. Furthermore, Pf-IRBC has been shown to induce the NF-*κ*B-regulated inflammatory pathway in human cerebral endothelium [83]. Also, curcumin has been shown to reduce the production of proinflammatory cytokines (TNF, IL-12, and IL-6) *in vitro*, and inhibition of iNOS by curcumin suppresses the production of IFN-*γ* and IL-12. iNOS has been shown to mediate ROS production, which is cytotoxic against *Plasmodium* [84]. Furthermore, curcumin can upregulate heme oxygenase-1 (HO-1) gene and protein expression by protecting brain endothelial cells from peroxide-mediated toxicity and toxicity due to free heme [85]. Another study reported that curcumin suppresses activation of C-Jun *N*-terminal kinases (JNK), which belongs to the mitogen active kinase family (MAP kinase) and is activated in response to inflammatory cytokines and stress conditions [2, 86]. Its activation induces a transcription-dependent apoptotic signaling pathway, resulting in cell death during experimental cerebral malaria (CM) [39, 86]. In a murine model of CM, curcumin administration resulted in a partial reduction of CM and delayed death [66]. Interestingly, curcumin has been shown to suppress proinflammatory cytokine responses and provide protection to endothelial cells.

The pathogenesis of malaria is determined by the interaction between *P. falciparum* and human host cells. *P. falciparum* infection can develop into severe malaria, even CM, associated with sequestration of *P. falciparum-infected erythrocytes blood cells* (Pf-IRBC) in the brain, causing coma [87]. Pf-IRBC is known to play a role in the apoptosis of bEnd. Three cells are amplified by parasitemia levels and incubation period [39]. The increase in the apoptosis of bEnd.3 cells depends on the synergy between parasitemia, host cells, platelets, and peripheral blood mononuclear cells (PBMC) [39]. The apoptotic mechanism of brain endothelial cells induced by Pf-IRBC is associated with the cytoadherence of Pf-IRBC. Pino et al. [84] revealed that the cytoadherence of Pf-IBRC modulated brain endothelial expression of the TNF-*α* superfamily genes, apoptosis-related genes (Bad, Bax, caspases, and iNOS) and activated the Rho-kinase signaling pathway that induces the production of ROS by endothelial cells that cause cell death. Several possible mechanisms cause endothelial cell dysfunction, including sequestration and adhesion-independent mechanisms [39]. Curcumin (IC50:10 *μ*M) inhibited the growth of *P. falciparum* and was able to protect endothelially, by reducing apoptosis of bEnd.3 cells, with Pf-IRBC, platelets, and PBMC. These findings suggest that curcumin is a potential adjunctive therapy for treating CM in the future.

### 3.3. Piperine as an Antiplasmodium

Only five articles (Table 2) discussed piperine as antiplasmodium despite black pepper (*Piper nigrum*) being long used as a traditional medicine for malaria. However, the number of publications is likely to increase as research trends develop. Piperine is a potent antioxidant and has been reported in many experimental models of cancer [89]. Piperine exhibits a higher antioxidant potential compared to vitamin E, attributed to its strong hydrogen-donating ability, metal chelating capacity, and effectiveness to scavenge free radicals, mainly ROS [90]. During malaria infection, both the host and parasites are under oxidative stress, with ROS (e.g., superoxide anions and hydroxyl radicals) produced by activated neutrophils in the host and during hemoglobin degradation in parasites. The effects of ROS on malaria can be both beneficial and pathological, depending on the amount and location of production. *Piper nigrum* has been used by South Indian traditional healers to treat fevers in general, malaria, asthma, cold, intermittent fever, cholera, colic pain, and diarrhea [91, 92]. Kamaraj et al. [38] reported that the ethyl acetate seeds extract of *Piper nigrum* showed promising *in vitro* antiplasmodial activity against *P. falciparum* 3D7 and INDO strains with IC_50_ values of 12.5 and 12.0 *μ*g/mL, respectively, with low cytotoxicity (TC50 = 87.0 g/mL). Furthermore, the significant therapeutic index of 7.0 in alkaloids piperine, guineensine, piperidine, N-feruloyltyramine, and N-isobutyl-2E, and 4E-dodecadienamide have been isolated from *Piper nigrum,* and piperine has been reported as a stimulator of *in vitro* melanocyte proliferation [93]. Also, an ethnobotanical survey of twenty traditional healers in India reported that *Piper nigrum* was used in decoction form for malaria chemoprophylaxis [33]. In 2013, Thiengsusuk et al. researched 27 medicinal plants in Thailand, including *Piper chaba* Hunt (the active compound is piperine), showing that the extract *Piper chaba* Hunt showed potent antimalarial activity IC_50_: <10 *μ*g/ml [88]. Furthermore, piperine IC_50_: 111.5 *μ*M and IC_90_: 329 *μ*M change parasite (3D7 *P. falciparum*) morphology after 48 hours of exposure. Specifically, morphological changes (cytoplasm condenses) start at 8 hours, but effects were observed after 12 hours of piperine exposure compared to untreated cells, slowing the growth of some surviving parasites. At IC_90_, almost all parasites died after 8 hours of exposure to piperine, suggesting that the window of activity is likely to be the late ring to trophozoite stages (8–12 h) [40]. However, there were no effects of piperine observed on modulating (inducing or inhibiting) the expression of all *P. falciparum* resistance genes under investigation including *Plasmodium falciparum multidrug resistance 1* (pfmrp1), *Plasmodium falciparum multidrug resistance protein 1* (pfmdr1), and *Plasmodium falciparum chloroquine resistance transporter* (pfcrt) [40], implying a low risk of development of resistance development to piperine of *P. falciparum*. In a recent study, *Piper nigrum* (IC50: 16.25 and 20.26 *μ*g/mL) showed promising antimalarial activity against insensitive and resistant *P. falciparum* (FCK2 and INDO) strains in inhibiting *Plasmodium lactate dehydrogenase* (PfLDH) [10]. However, the mechanism of action of piperine at molecular and cellular level remains unclear.

### 3.4. The Potential Use of a Curcumin-Piperine Combination as an Antimalarial Agent

Based on our understanding from various studies, curcumin has already shown great potential against *Plasmodium* spp, both *in vitro* and *in vivo* [28, 41]. Nevertheless, its poor bioavailability and also rapid metabolism are issues to overcome to exploit the full benefits of this plant-derived compound [8]. Bioenhancers such as piperine, extract from black pepper *(Piper nigrum)* can improve the bioavailability of curcumin by 2000-fold [8, 94]. Martinelli et al. [28] evaluated the effect of curcumin-artemisinin combination against an artemisinin-resistant clone of *P. chabaudi*. Also, they tested the efficacy of piperine in increasing the bioavailability of curcumin, thus increasing its efficacy [95]. The study showed that oral administration of 300 mg/kg BW of curcumin combined with 20 mg/kg BW of piperine and 150 mg/kg of artemisinin had no conclusive effect on the course of infection. However, the peak parasitemia and antimalarial activity reached by the curcumin and curcumin/piperine treatment groups were significantly lower than the control untreated group [28].

**Figure 2:**
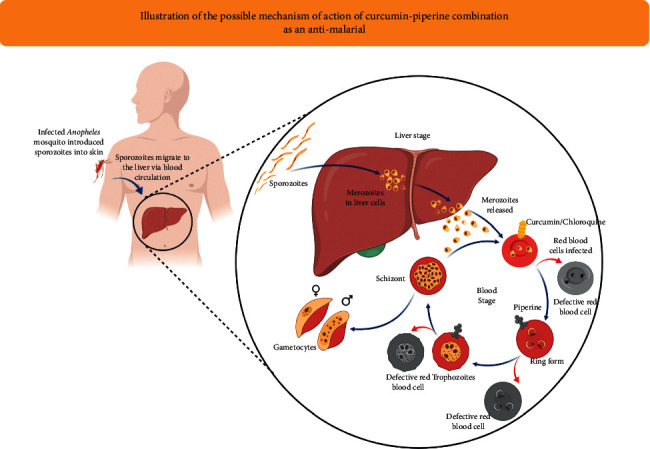
Illustration of the possible mechanism of action of curcumin-piperine combination as an antimalarial. Infected Anopheles mosquito introduced sporozoites into skin. Sporozoites migrate to liver via blood circulation and initiate the liver stage. At the liver stage, sporozoites invade the hepatocyte and undergo further development into merozoites. At the blood-stage, merozoites infect RBCs and start degrading hemoglobin (Hb). Heme released is polymerized to curtail its toxicity on the parasite. For example, chloroquine (medication primarily used to prevent and treat malaria) kills the parasites by blocks heme polymerization. Curcumin, probably having a similar action with chloroquine, restricts parasite development at the early stage. Meanwhile, piperine can make morphological changes (cytoplasm condenses) at the late ring to trophozoites stages, thus becoming defective red blood cells. Piperine as a bioenhancer may potentiate the effects of curcumin. Hence, combining curcumin and piperine as an antimalarial is expected to act at an earlier stage of the blood stage.

**Figure 3:**
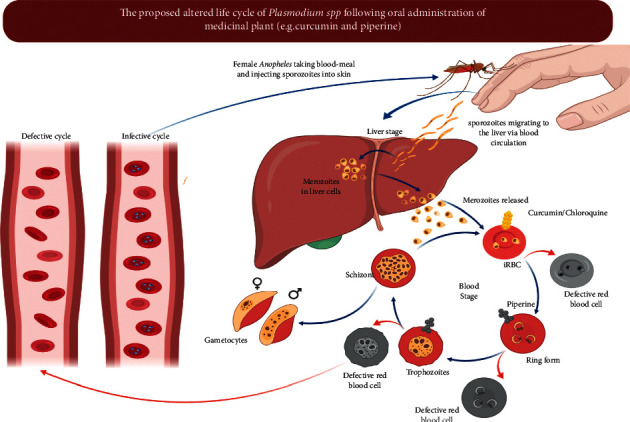
The proposed modification of the *Plasmodium* life cycle. The navy-colored arrows represent the normal infective life cycle, while the red-colored arrows represent the defective life cycle due to the action of the curcumin-piperine combination. There is a possibility that the parasite development was disrupted at the initial or late stages of red blood cells (defective red blood cells), so it cannot infect other red blood cells.

**Figure 4:**
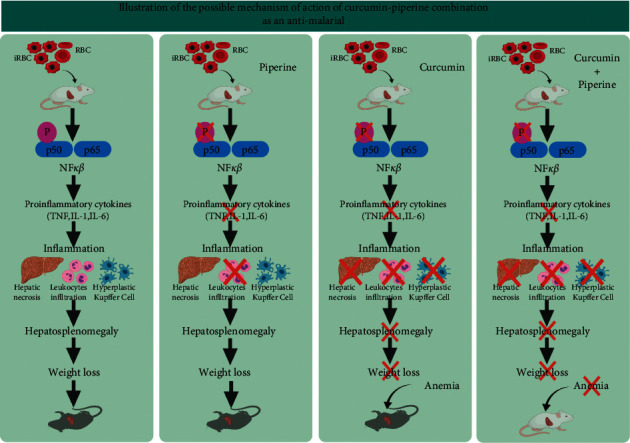
An illustration to explain the mechanism of action of curcumin-piperine combination as an antimalarial in animal models. When Swiss mice are infected with *P. berghei* ANKA strain, they show malaria symptoms and die between 8 and 12 days. Piperine alone inhibits phosphorylation of NF kappa B prevents leukocyte infiltration, but hepatic necrosis and hyperplasia of Kupffer cells remain visible. The animals eventually die due to parasite build up, causing hepatosplenomegaly and weight loss. Curcumin alone is also known to inhibit the phosphorylation of NF kappa B preventing leukocyte infiltration, hepatic necrosis, and hyperplasia of Kupffer cells. Thus, hepatosplenomegaly and weight loss are not seen. However, the animal eventually died by almost 20 days due to parasite build up and anemia. However, if the animals are given piperine and curcumin combination, the parasites are cleared and the animals are completely protected against mortality. Thus, while curcumin counteracts the inflammatory response, piperine potentiates the effects of curcumin, making this combination as a potential therapy for preventing malaria.

Furthermore, Neto et al. [68] evaluated the efficacy and the drug interactions between curcumin/piperine/chloroquine with curcumin/piperine/artemisinin in *P. chabaudi* parasites resistant to chloroquine (AS-3CQ) and artemisinin (AS-ART). Also, they verified the effects of curcumin, chloroquine, and artemisinin drug treatment on the UPS (ubiquitin/proteasome system), showing that the interaction between curcumin/piperine/chloroquine was additive, reducing the parasite load seven days after treatment. Interestingly, although both drugs have different structures and modes of action, they both have anti-inflammatory properties which possibly contribute to the reduction in parasitemia [70]. Curcumin is known for its immunomodulatory properties, including activation of TLR2, increased IL-10, and production of antiparasite antibodies [70]. Chloroquine is well known for its antimalarial schizonticidal activity and its anti-inflammatory properties such as inhibition of TNF-*α*, IL-1*β*, and IL-6, making both drug combinations attractive for the treatment of other diseases where an excess of proinflammatory cytokines is produced. It is believed that curcumin is a potential compound for adjunctive treatment of CM, which is often treated with quinine (chloroquine derives) [30]. However, the curcumin/piperine/artemisinin combination did not show a favorable drug interaction in this murine model of malaria [68]. Based on the mechanism of action of curcumin and piperine that has been discussed previously, it is likely that most parasite development is impaired at the blood stage. Meanwhile, at the liver-stage, plasmodia infection becomes very limited to trigger an immune response to the liver stage. Although curcumin and piperine are known to act at different phases, it is hypothesized that the combination of curcumin and piperine can attenuate plasmodia in the early stages of the blood stage (Figure 2), increasing the immune response to malaria liver-stage infection, which implies increased protection (Figure 3). This phenomenon prompts us to think that the combination of curcumin and piperine significantly reduces the likelihood of developing severe clinical manifestations of malaria (i.e., inflammation, hepatosplenomegaly, and anemia) (Figure 4). The combination of curcumin and piperine is expected to be a potential candidate in the development of future antimalarial drugs.

## 4. Conclusion

The data presented in this review demonstrates the potential combination of curcumin and piperine (therapeutic and prophylactic) as a candidate antimalarial drug. Curcumin has many pharmacological activities, with antioxidant, anti-inflammatory, and antiapoptotic properties. Piperine is a potent antioxidant and a bioenhancer that may potentiate the effect of curcumin, especially by producing ROS which is cytotoxic against malaria parasites. Combining these compounds is likely to have the same effect as chloroquine that attenuate and restrict the development of parasites. A comprehensive approach is also needed to evaluate the specific mechanism of action of these compounds as antimalarial agents. For further large-scale development, research related to evaluating the potential for the combination of curcumin and piperine is urgently needed [96].

## Figures and Tables

**Table 1:** Several studies related to curcumin as an antiplasmodium.

No	Activities	Subject	Type of study	Findings/outcomes	References
1	Antioxidant	Chloroquine-resistant *P. falciparum* and *P. berghei*-infected mice	*In vitro*	Curcumin (IC_50_:5 *μ*M) inhibits chloroquine-resistant *P. falciparum* growth in culture	[27]
			*In vivo*	Curcumin (100 mg/kg BW) reduces blood parasitemia by 80–90% and significantly enhances their survival	
2		*P. berghei-*infected mice	*In vitro*	Curcumin and artemisinin combination show an additive interaction in killing *P. falciparum*	[41]
			*In vivo*	Three oral dependent doses of curcumin following a single injection of alpha, beta-arteether can inhibit recrudescence due to alpha, beta-arteether monotherapy and also ensure almost 100% survival rate of the animal models.	
3		CQS (3D7 and D10) and CQR strains (Dd2 and 7G8) of *P. falciparum*	*In vitro*	Curcumin (25–100 *μ*M) caused specific inhibition of PfGCN5 HAT	[42]
4		*P. berghei ANKA-infected mice*	*In vivo*	The combination of these two herbal drugs (AP + CUR, HC + CUR) inhibited the ring stage of the parasite with no *in vivo* toxicity	[43]
5		*P. falciparum* 3D7 line was cultured in human 0^+^ erythrocytes	*In vitro*	Curcumin inhibited *P. falciparum* glyoxalase (GloI)	[44]
6		*-*	Docking and *in silico* ADMET	Curcumin inhibits PfSAHH *(Plasmodium falciparum S-adenosyl-L-homocysteine hydrolase)*	[45]
7		*P. berghei*-infected mice and murine RAW 264.7 macrophages	*In vivo*	Encapsulation of curcumin in PLGA increased parasite suppression by 56.8% at 5 mg/kg of nanoformulation, which was higher than free curcumin (40.5%) at 10 mg/kg	[46]
			*In vitro*	The IC_50_ of Cur-PLGA (292.6 *μ*g/mL) was lower than free curcumin (1000 *μ*g/mL)	
8		*P. falciparum*	*In silico* simulation study	Curcumin shows a high affinity for binding with HGPRT of PfHGPRT as virulence factors in malaria progression	[47]
9		*P. vivax* was cultured in RPMI 1640 culture medium (with 10% human serum and gentamycin 2 *μ*g/ml) at 37°C in a 5% CO_2_ incubator	*In vitro*	Ethanol extracts of *Curcuma caesia* and *Curcuma longa* showed significant parasitemia inhibition ranging from 5.875.6% and 2–29.8% against *Chloroquine-resistant P. vivax*	[48]
10		Chloroquine-resistant *P. falciparum* INDO strain and *P. berghei* (ANKA) infected BALB/c mice	*In vitro*	Curcumin-loaded in FΔF nanotubes showed *P. falciparum* inhibition (IC_50_, 3.0 *μ*M) compared to free curcumin (IC_50_, 13 *μ*M)	[49]
			*In vivo*	Ccm-FΔF (equivalent to 50 mg/kg BW of curcumin) significantly decreased parasitemia and increased life span compared to free curcumin	
11		Chloroquine (CQ) sensitive strain of *P. yoelii* (N-67)	*In vitro*	Curcumin-bound chitosan nanoparticles can traverse the mucosal barrier intact and inhibited parasite lysate in a dose-dependent manner, with a lower IC_50_ value than chloroquine.	[29]
			*In vivo*	Curcumin bound to chitosan nanoparticles (1 mg) shows 100% survival	
12		3D7 (chloroquine-sensitive strain) and *P. berghei* (ANKA) infected C57BL/6 mice	*In vitro*	Nanotized curcumin (IC_50_: 0.5 *μ*M) inhibited ten-fold more *P. falciparum* than its native counterpart (IC_50_: 5 *μ*M)	[50]
			*In vivo*	Nanotized curcumin (20 mg/kg BW and 4 0 mg/kg BW) prolonged the survival of mice by more than 2 months with complete clearance of parasites compared to the untreated animals	
13		Chloroquine-sensitive 3D7 (West Africa) and chloroquine-resistant RKL-2 strain (Raurkela, Orissa, India) of *P. falciparum*	*In silico*	Curcumin analog showed various functional groups of curcumin and its analogs against the PfATP6 protein	[51]
			*In vitro*		
14		Sensitive 3D7 strain of *P. falciparum*	*In vitro*	50 *μ*g/mL of six curcumin derivates showed 100% schizont inhibition	[52]
15		3D7 chloroquine-sensitive strain of *P. falciparum*	*In vitro*	Curcumin (5 *μ*M) produced ROS which induced cytotoxicity and disrupted plasmodium microtubule stabilization, schizogony, and apicoplast segregation	[31]
16		*P. falciparum* drug-susceptible 3D7 clone of the NF54 isolate and the K1 strain (chloroquine and pyrimethamine resistant)	*In vitro*	Curcumin (10 *μ*M) induced intracellular ROS production resulting in PPAR*ɣ*/Nrf2 activation, increasing CD36 expression in monocytes/macrophages for phagocytosis of infected red blood cells	[30]
17		—	*In vitro*	Curcumin (0.4 mM) inhibits formation of *β*-hematin with an efficiency of 78.8% compared to amodiaquine (91.8%) and DMSO (10.7%)	[13]
18		A chloroquine-resistant strain of *P. falciparum* (MRC-pf-303) cultured in human O^+^ washed erythrocytes and *P. berghei ANKA-infected mice*	*In vitro*	Curcumin (IC_50_:17.4 *μ*M) inhibited parasites at their ring stage	[53]
			*In vivo*	Andrographolide-curcumin reduced parasitemia (29%) compared to the control (81%), as well as prolonged life span 2-3 fold	
19		Chloroquine (CQ) sensitive (D6 clone) and CQ-resistant (W2 clone) strains of *P. falciparum*	*In vitro*	Curcuminoids (IC_50_: 2 *μ*M) inhibited PfTrxR	[54]
20		Chloroquine-sensitive (CQ-S) and chloroquine-resistant (CQ-R) *P. falciparum*	*In vitro*	Several curcumin analogs effectively inhibited *P. falciparum* growth compared to curcumin. The most potent curcumin compounds 3, 6, and 11 were inhibitory for CQ-S *P. falciparum* at IC_50_ of 0.48, 0.87, 0.92 *μ*M and CQ-R *P. falciparum* at IC_50_ of 0.45 *μ*M, 0.89 *μ*M, 0.75 *μ*M, respectively	[55]
21		*P. falciparum* recombinant PfGST isolated from *E. coli* cells	*In vitro*	Curcumin inhibits PfGST with IC_50_: 69 *μ*M	[56]
22		*P. yoelii-infected mice*	*In vivo*	Curcumin-loaded eudragit-nutriosomes increased the survival of malaria-infected mice relative to free curcumin-treated control	[57]
23		*P. falciparum* chloroquine-resistant (W2) and chloroquine-sensitive (3D7) strains were maintained in continuous culture using human RBCs	*In vitro*	Coencapsulated NCs exhibited a significant reduction in *P. falciparum* parasitemia, better than QN/CR, and prolonged survival rate	[58]
			*In vivo*		
24		*P. berghei NK-65* infected mice	*In vivo*	Both nano encapsulated artemisinin (50 mg/kg/day) and artemisinin plus curcumin (100 mg/kg/day) cured all malaria-infected mice within the same postinoculation period	[59]
25		—	Molecular docking	The binding of curcumin and its analogs to Ca (2+) ATPase (PfATP6) of *P. falciparum* (the target of many antimalarial drugs) is mediated by both hydrophobic and polar interactions	[60]
26		*P. berghei-*infected mice	*In vivo*	Nanotized conjugate curcumin formulation can prolonge life span 90 days with complete eradication of the parasites from RBC	[61]
27		*P. falciparum* (intraerythrocytic forms, strain NF54).	*In vitro*	Curcuma exhibited high activity (IC_50:_<2.5 *μ*g/mL) against parasites of the genera *leishmania, trypanosoma, and Plasmodium*	[7]
28		*P. berghei-*infected mice	*In vivo*	Curcuminoid-loaded liposomes (40 mg/kg BW) along with *α*/*β* arteether (30 mg/kg BW) cured infected mice and prevented recrudescence	[62]
29		*P. berghei-*infected mice	*In vivo*	Curcumin-nanostructured lipid carriers (Cur-NLC) was significantly higher compared with that of free cur at the dose of 40 mg/kg/day	[63]
30		*P. berghei*	*In vivo*	CA-PLGA nanoparticle 5 and 10 mg/kg doses. The drug efficacy was determined on day 5 and 8.	[64]
31		*P. berghei-*infected mice	*In vivo*	A combination of 35 mg/kg of curcumin along with either 5 mg/kg or 1 mg/kg BW of PRI demonstrated 100% antimalarial activity and survivability beyond 20 days	[65]
32		*P. berghei ANKA*-infected mice	*In vivo*	Curcumin 50 mg/kg/day reduced parasitemia and increased the survival rate	[66]
33		*P. berghei-*infected mice	*In vivo*	Curcumin 100 mg/kg BW showed a 2-fold increase in the survival period (15–21 days) compared to those treated with the free curcuminoids at the same dose	[67]
34		ART-resistance clone of *P. chabaudi*	*In vivo*	Curcumin 300 mg/kg/day and piperine 20 mg/kg/day had only a modest antimalarial effect and could not reverse the artemisinin-resistant phenotype	[28]
			*In vitro*		
35		*P. chabaudi-*infected mice	*In vivo*	Curcumin 500 mg/kgBW, piperine 20 mg/kgBW and choloroquine 2.5 mg reduced parasitemia to 37% seven days after treatment compared to the control group 65%	[68]

36	Anti-inflammatory	*P. berghei ANKA-*infected mice	*In vivo*	PLGA-curcumin (5 mg/dose providing 350 *μ*g of curcumin) was 15-fold lower in preventing the breakdown of blood-brain barrier and inhibition of brain mRNAs for inflammatory cytokines, the chemokine receptor CXCR3 and its ligand CXCL10, with an increase in the inflammatory cytokine IL-10	[69]
				PLGA-curcumin inhibiting the sequestration of parasitized-RBCs and CD8^+^T cells in the brain	
37		*P. berghei-*infected mice	*In vivo*	Curcumin *α*, *β* arteether combination (5 mg + 750 *μ*g) prevents recrudescence through immunomodulation in *P.berghei-*infected mice	[70]
38		*P. berghei-*infected mice	*In vivo*	Curcumin 5 mg reversed all parameters: Inflammatory responses, CD8^+^ T cell, and pRBC sequestration into the brain and blood-brain barrier (BBB) breakdown	[71]
39		*P. berghei ANKA-*infected mice	*In vivo*	Curcumin and lipid-based drug delivery systems (LBDDSs) combined with *β*-arteether (30 mg/g) reduced cytoadherence and subsequent parasite sequestration of parasite-infected erythrocytes by inhibiting NF-kB activation, thereby suppressing proinflammatory cytokine responses and expression of adhesion molecules in endothelial cells	[72]
40		*P. berghei NK65-*infected rat	*In vivo*	Curcumin 30 mg/kg BW involved inhibition of GSK3*β*	[73]

41	Antiapoptotic	*P. falciparum* chloroquine-resistant strain (TM 267)	*In vitro*	Curcumin IC_50_ ∼10 *μ*M decreased bEnd.3 apoptosis to 60.0% and 79.6% upon pretreatment and cotreatment, respectively, with Pf-IRBC, platelets, and PBMC	[74]

**Table 2:** Several studies related to piperine as an antiplasmodium.

No	Activities	Subject	Type of study	Findings/Outcome	Reference
1	Antioxidant activity	*P. falciparum* of FCK2 and INDO strains	*In vitro*	*Piper nigrum* exhibited antiplasmodial activity at IC_50_ <50 *μ*g/mL in *P. falciparum* lactate dehydrogenase (PfLDH) inhibition assay	[10]
2		Chloroquine (CQ) sensitive 3D7 and CQ-resistant INDO strains of *P. falciparum*	*In vitro*	*Piper nigrum* IC_50_:12.5 *μ*g/mL showed antiplasmodial activity	[38]
3		K1 (chloroquine-resistant) *P. falciparum* and 3D7 (chloroquine-sensitive)	*In vitro*	Piperine IC_50_: 111.5 and 59 *μ*M changes parasite morphology after 48 hours of exposure and has a low risk of resistance	[40]
4		K1 (chloroquine-resistant) *P. falciparum* and 3D7 (chloroquine-sensitive)	*In vitro*	The extract piper chaba hunt showed potent antimalarial activity IC_50:_ <10 *μ*g/ml	[88]

5	Chemoprophylaxis	Human	*Survey*	*Piper nigrum* were used in decoction form for malaria chemoprophylaxis	[33]

## Data Availability

The data supporting this review article are from previously reported studies, which have been cited.
